# Frailty syndrome among elderly and associated factors: comparison of two cities*

**DOI:** 10.1590/1518-8345.2897.3100

**Published:** 2018-11-29

**Authors:** Rosalina Aparecida Partezani Rodrigues, Jack Roberto Silva Fhon, Maria de Lourdes de Farias Pontes, Antonia Oliveira Silva, Vanderlei José Haas, Jair Lício Ferreira Santos

**Affiliations:** 1 Universidade de São Paulo, Escola de Enfermagem de Ribeirão Preto, Centro Colaborador da OPAS/OMS para o Desenvolvimento da Pesquisa em Enfermagem, Ribeirão Preto, SP, Brasil.; 2 Universidade Federal da Paraíba, Departamento de Enfermagem, João Pessoa, PB, Brasil.; 3 Universidade Federal da Paraíba, Instituto Paraibano de Envelhecimento, João Pessoa, PB, Brasil.; 4 Universidade Federal do Triângulo Mineiro, Uberaba, MG, Brasil.; 5 Universidade de São Paulo, Faculdade de Medicina de Ribeirão Preto, Ribeirão Preto, SP, Brasil.

**Keywords:** Frailty, Aged, Aging, Geriatric Nursing, Comparative Study, Health of the Elderly

## Abstract

**Objective:**

to compare the frailty syndrome among elderly people living at home in two Brazilian cities and to identify factors related to sociodemographic and health-related variables.

**Method:**

population-based cross-sectional study with 480 elderly individuals from the cities of Ribeirão Preto/SP and João Pessoa/PB, with application of the Mini Mental State Examination instruments and the Edmonton Frailty, Geriatric Depression and Lawton and Brody scales. Descriptive analysis, Chi-square test, Fisher’s test, Student’s t-test, Spermann’s correlation and Logistic regression were used. In all analyzes, the level of significance was set at p≤0.05.

**Results:**

in relation to frailty, it was verified that living in Ribeirão Preto, presenting advanced age, low schooling, multiple chronic diseases, reduced cognitive status and functional capacity, besides depressive symptoms, are factors associated with the frailty syndrome, in both cities.

**Conclusion:**

we identified that the frailty syndrome in the elderly of both cities has a relation with the place where the elderly person lives, age, schooling, number of diseases, reduction of cognitive status, functional capacity and presence of symptoms depressive.

## Introduction

In a global context of population aging, in Brazil, there has been an accelerated increase in the proportion of the elderly. In 2010, according to a survey by the Brazilian Institute of Geography and Statistics (IBGE), there were a total of 23.5 million people aged 60 or over, and by 2013 this number reached 64.8 million. It is also noted an increase in life expectancy, with projections that reach, by 2041, 80 years^(^ ^1^ ^)^ .

The aging process is accompanied by a set of physiological, psychological and social changes that can trigger multiple syndromes, among them the frailty^(^ ^2^ ^)^.

Frailty is a state of multidimensional change in which there is increased vulnerability and loss of resistance to external stressors, which increases the chance of certain adverse health events such as decreased strength, endurance and physiological function^(^ ^3^ ^)^. Under these conditions, the risk of dependence and/or death is high^(^ ^4^ ^)^.

In order to define frailty, the Canadian Initiative on Frailty and Aging (CIF-A) elaborated the concept based on a holistic approach determined by biological, psychological and social factors related to the life trajectory of the elderly^(^ ^5^ ^-^ ^6^ ^)^.

In a systematic review with 19 articles, the authors verified the prevalence of frailty in the elderly between 2.3% and 75.0%^(^ ^7^ ^)^. Research indicates that frailty is associated with demographic factors, such as female sex and old age^(^ ^7^ ^-^ ^8^ ^)^, and with clinical conditions, such as decreased functional capacity and cognitive status^(^ ^9^ ^)^, increased number of falls and hospitalizations^(^ ^10^ ^)^ and death^(^ ^11^ ^)^.

In Brazil, there are few studies on the subject, especially those comparing two cities that can be influenced by the inequalities present in the different Brazilian regions, mainly life expectancy, low schooling, monthly income, housing and health conditions of the elderly, and conflicting house arrangements, in addition to suffering difficulties in the attention to healthcare.

Thus, in view of the accelerated aging process and the importance of investigating situations that contribute to frailty in the elderly, this study aims to compare the frailty syndrome among elderly people living at home in two Brazilian cities and to identify factors related to sociodemographic and health variables.

## Method

This is an analytical and cross-sectional study carried out in the cities of Ribeirão Preto, located in the Southeast region, northeast of the state of São Paulo, and João Pessoa, capital of the Paraíba state, located in the Northeast of Brazil. Both have a similar number of inhabitants: 682,302 and 811,598, respectively^(^ ^12^ ^)^.

The following criteria were defined for participation in the study: being 60 years of age or older, of both sexes, living in the urban area and being able to respond to the instruments of data collection. The exclusion criteria were elderly with neurological diseases and that after the first contact did not attend the researchers in up to three consecutive visits.

For the identification of the elderly, a sample was chosen by double-stage conglomerate. In the first, the sample unit was considered the census sector and, in the second, age over 60 years. The sample was calculated in each city to guarantee maximum error of 6.3% with 95% probability, in situation of maximum variability. To reach the value of 240 elderly people in each city to compose the sample, we planned to raffle 20 sectors in both cities and to ensure the sample self-weighting, for which we fixed the number of 12 elderly people by census sector. Once the sector was chosen, a new lottery of the streets and blocks was made to identify the elderly people from the starting point, clockwise.

The data were collected in the two cities by teams composed of undergraduate and graduate students previously trained by the study coordinator in both cities to apply the different instruments. The elderly were contacted in 2014, in their respective homes, and the interviews occurred in 2015, after previous scheduling, with a duration of approximately 45 minutes each. To this end, the following instruments were applied:

a)Sociodemographic profile. For the characterization of participants, the variables were: sex (male and female); age (in complete years); marital status (with and without partner); family arrangement (alone, spouse, relatives and others); schooling (years of formal study); income (retirement, other sources of income and without retirement) and self-reported morbidities.

b)Mini Mental State Examination (MMSE). It is an instrument^(^ ^13^ ^)^ translated and validated into the Portuguese language^(^ ^14^ ^)^ whose score is related to the educational level of the respondents. The questions are grouped into seven categories and each of them evaluates different and specific cognitive functions: orientation on time (5 points); orientation on place (5 points); 3-word registration (3 points); attention and calculation (5 points); word recall (3 points); language (8 points); and visual constructive capacity (1 point). The score can vary from 0 to 30 points, and the cutoff points are: 13 for illiterate, 18 for low/medium schooling and 26 for high schooling.

c)Edmonton Frail Scale (EFS), prepared by the Canadian Initiative on Frailty and Aging (CIF-A)^(^ ^15^ ^)^ and validated for the Portuguese language^(^ ^16^ ^-^ ^17^ ^)^. The scale evaluates nine domains represented by 11 items: a) cognitive area with the clock test (1 item); b) general health status (2 items); c) functional independence (1 item); d) emotional support (1 item); e) medication use (2 items); f) nutrition (1 item); g) mood (1 item); h) continence (1 item); i) functional performance Time Up and Go for balance and mobility (1 item). The maximum score is 17 points and represents the highest level of frailty.

d)Geriatric Depression Scale (GDS), reduced version of the original scale^(^ ^18^ ^)^ and validated for the Portuguese language^(^ ^19^ ^)^, with dichotomous responses. The score ranges from zero (absence of depressive symptoms) to 15 points, with the cutoff point> 5 indicating the presence of symptoms.

e)Lawton and Brody scale, which evaluates the Instrumental Activities of Daily Living (AIVD)^(^ ^20^ ^)^, adapted to the Brazilian context^(^ ^21^ ^)^, and encompasses more complex social activities. The score ranges from seven (highest level of dependence) to 21 points (complete independence). It is considered independent the elderly capable of performing all IADL without help and dependent the one who needs help, who will be categorized as partially dependent or totally dependent.

The information was stored in a Microsoft Excel^®^ worksheet. To avoid errors, we performed double typing followed by corrections. Subsequently, the information was imported into the statistical program Statistical Package for the Social Sciences - SPSS v. 22.

In the analysis of the quantitative variables, the descriptive analysis was carried out and we chose to use the measures of central tendency (mean and median) and dispersion (standard deviation). For the categorical variables, absolute and relative frequencies were adopted.

In the bivariate analysis, with the city as an independent variable (Ribeirão Preto and João Pessoa), the chi-square test was used for sociodemographic variables (sex, age group, marital status, income and family arrangement). In order to compare the means between the two cities and sociodemographic variables and between the sexes (male and female) with the clinical variables (cognitive status, frailty, functional capacity and depressive symptoms), we applied the Student’s t Test and the correlation Spearmann score between the frailty score and the number of diseases.

For the final analysis, Multiple Linear Regression was used, with frailty as the dependent variable, including the cities (Ribeirão Preto and João Pessoa), sex (male and female), age, schooling, number of diseases, MMSE (with and without cognitive deficit), IADL and GDS. In all analyzes, the level of significance was set at p ≤ 0.05.

The research was evaluated and approved by the Ethics and Research Committee of the School of Nursing of Ribeirão Preto, University of São Paulo, under the Approval Certificate (CAAE) number 47155115.3.0000.5393.

## Results

Of the 480 (100%) elderly, there was a predominance, in both cities, of elderly women, aged 80 years or more and retirees. In relation to the marital status, in Ribeirão Preto, there was prevalence of elderly with a partner and, in João Pessoa, without a partner. As for the family arrangement, in Ribeirão Preto, most elderly lived only with their spouses (29.6%) and in João Pessoa, with spouse, children and grandchildren (28.0%) (Table 1).

**Table 1:** Sociodemographic profile of elderly participants in Ribeirão Preto, SP and João Pessoa, PB, Brazil, 2015*

Variable	Category	Ribeirão Preto		João Pessoa		p*^†^
		n	%	n	%	
Sex	Male	91	37.9	74	30.8	0.10
	Female	149	62.1	166	69.2	
Age	60 – 64	44	18.3	48	20.0	0.93
	65 – 69	45	18.8	43	17.9	
	70 – 74	49	20.4	48	20.0	
	75 – 79	46	19.2	40	16.7	
	80 or more	56	23.3	61	25.4	
Marital status^‡^	With partner	141	59.0	101	42.3	<0.001
	Without partner	98	41.0	138	57.7	
Family arrangement^§^	Alone	34	14.2	18	7.5	<0.001
	With partner	71	29.6	22	9.2	
	Spouse, children, son-in-law or daughter-in-law	5	2.1	38	9.2	
	With children	15	6.3	21	8.8	
	Elderly, children and grandchildren	20	8.3	67	28.0	
	Elderly and spouse	0	0.0	3	1.3	
	With other elderly people	3	1.3	2	0.8	
	With grandchildren	0	0.0	3	1.3	
	Non-familial arrengement	54	22.5	58	24.2	
Income	Retirement	148	61.7	128	53.3	0.03
	Retirement and other sources	37	15.4	59	24.6	
	Other sources	55	22.9	53	22.1	

*Chi-square test; †p ≤ 0.05; ‡ §There was loss of information in the variables marital status and family arrangement of the elderly

In Joao Pessoa, women between 65 and 69 years old presented a mean score of frailty of greater magnitude in relation to men. Those who lived with their spouses reached a higher average in comparison with men who also lived with spouses (Table 2).

**Table 2:** – Mean values of the frailty score of elderly participants from Ribeirão Preto, SP and João Pessoa, PB according to sociodemographic variables and number of diseases. Brazil, 2015*

Variable	Ribeirão Preto		p^†^	João Pessoa		p^†^
	
	Female	Male		Female	Male	
			
	Mean (SD)	Mean (SD)		Mean (SD)	Mean (SD)	
Age group						
60 – 64	3.89 (1.60)	3.78 (1.39)	0.855	4.29 (1.96)	3.43 (2.53)	0.209
65 – 69	5.22 (2.86)	4.50 (2.46)	0.373	5.03 (2.47)	3.21 (3.14)	0.045
70 – 74	6.03 (3.39)	4.76 (2.75)	0.192	5.59 (3.19)	4.07 (3.32)	0.146
75 – 79	6.21 (2.41)	6.65 (3.16)	0.597	5.96 (3.00)	6.25 (2.90)	0.782
80 or more	8.27 (2.65)	7.31 (3.13)	0.220	6.95 (3.14)	7.25 (2.65)	0.715
Home Arrangement						
Alone	5.70 (3.37)	7.29 (3.30)	0.275	5.24 (2.54)	2.00 (-)	0.233
Spouse	5.00 (3.11)	5.46 (2.95)	0.525	6.83 (3.30)	3.80 (3.46)	0.048
With family	6.21 (2.80)	5.89 (3.21)	0.649	5.41 (2.93)	4.66 (3.15)	0.169
Non-familial arrangement	6.40 (2.71)	5.21 (2.88)	0.138	5.95 (2.97)	6.78 (3.14)	0.338
Income						
Retirement	5.99 (3.03)	6.04 (2.96)	0.913	6.10 (2.84)	5.24 (3.43)	0.131
Retirement and other sources	5.26 (2.73)	3.10 (2.77)	0.040	5.20 (3.34)	5.11 (3.32)	0.929
Other sources	6.08 (3.05)	5.57 (2.82)	0.639	5.14 (2.58)	3.70 (2.50)	0.116
Number of diseases						
Spearman Correlation^†^	0.488	0.500		0.445	0.608	
p<0.05	<0.001	<0.001		<0.001	<0.001	

*Student’s t-test; †p ≤ 0.05; ‡Spearman Correlation.

With regard to the cognitive status of the elderly according to sex, we found that in Ribeirão Preto, 137 (52%) had a cognitive deficit, with predominance among the female, 92 (35.1%), when compared to the male, 45 (17.2%). On the other hand, in João Pessoa, 55 (20.9%) had a cognitive deficit, being more prevalent in the female sex, 36 (13.7%), than in males, 19 (7.3%). In both cities, there was no statistical significance.

In Ribeirão Preto, there was a higher mean frailty score in females. In João Pessoa, the mean functional capacity and the presence of depressive symptoms were higher in females (Table 3).

**Table 3:** – Mean and median values of the frailty scores, functional capacity and depressive symptoms of elderly participants of Ribeirão Preto, SP and João Pessoa, PB according to sex. Brazil, 2015*

Variable	Ribeirão Preto		p^†^	João Pessoa		p^†^
	Female	Male		Female	Male	
Frailty						
Mean (=SD)	5.89 (2.98)	5.68 (3.04)	0.042	5.63 (2.93)	5.00 (3.29)	0.143
Median	6.00	6.00		5.00	4.50	
Functional capacity						
Mean (=SD)	18.38 (3.24)	17.46 (3.55)	0.573	17.23 (4.65)	15.84 (5.50)	0.045
Median	20.00	18.00		19.00	18.50	
Depressive symptoms						
Mean (=SD)	4.66 (3.40)	4.51 (3.24)	0.721	3.81 (2.98)	2.94 (2.42)	0.042
Median	4.00	4.00		3.00	2.50	

*Student’s t-test; †p ≤ 0.05

In the final model for the frailty scores, we found that, in the city of Ribeirão Preto, age, schooling, number of diseases, cognitive status, GDS score and IADL scores were statistically significant in relation to frailty (Table 4).

**Table 4:** Demographic and clinical factors associated with the frailty syndrome of elderly participants from Ribeirão Preto, SP and João Pessoa, PB, Brazil, 2015*

Variable	β^†^	Standard error	t	p^‡^
Ribeirão Preto	0.485	0.210	2.307	0.022
Male	-0.307	0.185	-1.661	0.097
Age	0.028	0.012	2.417	0.016
Schooling	-0.097	0.016	-5.986	<0.001
Number of diseases	0.359	0.034	10.660	<0.001
With cognitive deficit	0.931	0.209	4.446	<0.001
IADL^§^	-0.308	0.029	-10.670	<0.001
GDS^║^	0.184	0.030	6.227	<0.001

*Multiple Linear Regression; †β: Beta; ‡p≤0.05; §IADL: Instrumental Activities of Daily Life; GDS: Geriatric Depression Scale

In the categorical variables, it was verified that, for Ribeirão Preto, the frailty score presented higher average than that of the city of João Pessoa. In addition, the elderly with cognitive deficit had a higher average of frailty when compared to those who did not present a cognitive deficit (Table 4).

For the numerical variables, the positive values of the beta standardized regression coefficient indicate that the increase of the values of each predictive variable correlate with the increase of the frailty, whereas the negative coefficients show a decrease of frailty with the increase of the scores of the predictor variables (Table 4).

## Discussion

The frailty syndrome in the elderly of both cities investigated is related to the place where the elderly person lives, age, schooling, number of diseases, decrease in cognitive status and functional capacity and presence of depressive symptoms.

Age is a determining factor for the onset of this syndrome. In this study, it was observed that the risk of becoming frail increases over the years. A similar result was found in a study carried out in Turkey with 1,126 elderly people, in which the authors identified, in those aged 74-85, a greater likelihood of becoming frail, whereas in the group of elderly people over 85 this chance was 5.635 times greater^(^ ^22^ ^)^. Another study conducted in Brazil with 360 elderly individuals over 65 years of age found that individuals aged 80 years or older presented a 1.24 time higher risk of being frail when compared to those aged 65 to 79 years^(^ ^23^ ^)^.

The consequences and development of aging phenotypes are related to different domains, e.g. changes in body composition, imbalance between energy availability and demand, or deregulated signaling networks that maintain homeostasis and neurodegeneration with impaired neuroplasticity. These factors increase the susceptibility of the elderly, since they reduce the functional reserve, resistance to stress and make health unstable, which can trigger the frailty syndrome^(^ ^24^ ^)^.

The lower the schooling, the greater the risk of the elderly being considered frail. In Turkey^(^ ^22^ ^)^ and in Japan^(^ ^25^ ^)^, the authors found that having low levels of schooling is related to frailty, perhaps because the elderly with low schooling have little or no access to health information. Maintaining a healthy behavior through regular physical exercises and an adequate diet decreases the risk of being considered frail^(^ ^4^ ^)^.

Advancing age brings cognitive and physical losses, which can be aggravated by the accumulation of effects inherent to the aging process itself, as well as by the greater presence of chronic diseases^(^ ^26^ ^)^, such as cardiovascular diseases, cancer, Alzheimer’s disease, diabetes mellitus, arthritis and osteoporosis^(^ ^27^ ^)^.

In this research, it was verified that the greater the number of diseases presented by the elderly, the greater the probability that they will become frail. A survey conducted in Turkey with 1,126 elderly people identified a higher mean of chronic diseases among those considered frail (3.18 ± 1.72) compared to pre-frail (2.08 ± 1.36) and non-frail (1.78 ± 1.25), which indicates that it is a factor that increases the risk for this syndrome (p <0.001)^(^ ^22^ ^)^.

The data also showed a relationship between frailty and decreased cognitive status. Similar results were found in China in a study with 1,045 elderly. The authors verified that those with physical frailty presented 2.28 times more chances of developing cognitive impairment^(^ ^28^ ^)^, as observed in a Brazilian study whose results showed a negative correlation between both variables (r = -0.513, p <0.001)^(^ ^29^ ^)^.

Both frailty and decreased cognitive status are frequent conditions in the elderly, usually related to accumulation of molecular and cellular damage, hormonal and inflammatory dysregulation^(^ ^30^ ^)^. It is also common that they present neurological pathologies, cardiovascular diseases, decreased food intake, isolation and social vulnerability^(^ ^31^ ^)^. All these factors interact with each other and can influence the cycle of frailty and cognition^(^ ^32^ ^)^.

The IADL in the frail elderly showed an inverted relationship, that is, a lower functional capacity, which shows that the elderly are more likely to be frail. In Korea, results from a study of 196 elderly people showed that the frail elderly had greater limitations in the AIVD compared to those who were not frail (p <0.001)^(^ ^33^ ^)^.

In cases of frailty syndrome, there may be decreased muscle strength, reduced gait velocity and low tolerance to exercise. Consequently, there will be decreased secretion of the hormones estrogen, testosterone, luteinizing and dehydroepiandrosterone^(^ ^34^ ^)^. Physical inactivity is considered the fourth factor that increases the risk of being frail, only behind overweight, obesity, increased chronic diseases and death^(^ ^35^ ^)^. Promoting the regular practice of physical activity among frail elderly people can increase functional capacity and contribute to an active aging.

In the analysis of the elderly of both cities, those living in João Pessoa have a lower frailty score. This result may be due to the city being located in a coastal region, with several public facilities for guiding exercise practice, healthy nutrition and walks on the beach, different from Ribeirão Preto, which does not have these social facilities open to the public.

Another important result of this investigation has shown that elderly people with depressive symptoms are more likely to be frail. Data obtained from systematic reviews verified that the prevalence of both (depression and frailty) was of 38.60%^(^ ^36^ ^)^ and its co-occurrence was greater than 10%^(^ ^37^ ^)^. Both conditions may be related to the decline process, since depression is associated with decreased muscle strength and physical activity^(^ ^38^ ^)^. Physiologically, the mechanisms of both conditions are overlapping and involve changes such as subclinical vascular diseases that cause prefrontal white matter hyperintensities, chronic inflammation, oxidative stress, mitochondrial dysfunction, and hypothalamic-pituitary-adrenal axis (HPA) and interleukin 6 dysregulation ^(^ ^36^ ^)^.

It is important to point out that the present study has two limitations: the first one is related to the cross-sectional design adopted in both cities, which does not allow to infer the cause and effect between the variables investigated; and the second one concerns the comparison of the findings of the study with other studies, since there is no standardization of the instruments to evaluate this syndrome in the elderly, that is, it is a complex phenomenon that involves physical, psychological aspects, as well as social factors.

Thus, recognizing that frailty is a progressing syndrome with associated factors, it is the responsibility of health professionals to perform a more detailed evaluation in the various correlated aspects, with a view to interventions that can minimize it.

## Conclusion

The results of this study revealed a predominance of female elderly, over 80 years old and retired. In Ribeirão Preto, elderly people with a partner or who lived with the spouses prevailed. In João Pessoa, there was a predominance of the elderly without a partner and a family arrangement formed by other elderly, children and grandchildren.

Among the factors related to frailty, it was verified that living in Ribeirão Preto, presenting advanced age, low schooling, multiple chronic diseases, reduced cognitive status and functional capacity, and depressive symptoms are factors associated with the frailty syndrome.

The study contributes to the identification of the level of frailty in the elderly, which is a risk factor for the occurrence of falls, dependence, institutionalization and death. The objective of the evaluation is to identify the elderly’s deficits and prevent them, thus providing an improvement in the living conditions of this population.
